# Asexually propagated *Agave tequilana* var. azul exhibits variation in genetic markers and defence responses to *Fusarium solani*

**DOI:** 10.1093/aobpla/plac027

**Published:** 2022-06-08

**Authors:** Cristina Chávez-Sánchez, Norma Alejandra Mancilla-Margalli, Mayra Itzcalotzin Montero-Cortés, Federico Antonio Gutiérrez-Miceli, Guillermo Ariel Briceño-Félix, June Kilpatrick Simpson Williamson, Martín Eduardo Avila-Miranda

**Affiliations:** Postgraduate Studies and Research Division, Instituto Tecnológico de Tlajomulco, Tecnológico Nacional de México, Km 10 carretera Tlajomulco-San Miguel Cuyutlán, Tlajomulco de Zúñiga, CP 45640, Jalisco, México; Postgraduate Studies and Research Division, Instituto Tecnológico de Tlajomulco, Tecnológico Nacional de México, Km 10 carretera Tlajomulco-San Miguel Cuyutlán, Tlajomulco de Zúñiga, CP 45640, Jalisco, México; Postgraduate Studies and Research Division, Instituto Tecnológico de Tlajomulco, Tecnológico Nacional de México, Km 10 carretera Tlajomulco-San Miguel Cuyutlán, Tlajomulco de Zúñiga, CP 45640, Jalisco, México; Postgraduate Studies and Research Division, Instituto Tecnológico de Tuxtla Gutiérrez, Tecnológico Nacional de México, Carretera Panamericana Km 1080, Tuxtla Gutiérrez, CP 29050, Chiapas, México; Research and Technological Validation Program, Tequila Regulatory Council, A.C., Av. Patria 723, Zapopan, CP 45030, Jalisco, México; Department of Plant Genetic Engineering, CINVESTAV-IPN, Km 9.6 Libramiento Norte Carr. Irapuato-León, Irapuato, CP 36824, Guanajuato, México; Postgraduate Studies and Research Division, Instituto Tecnológico de Tlajomulco, Tecnológico Nacional de México, Km 10 carretera Tlajomulco-San Miguel Cuyutlán, Tlajomulco de Zúñiga, CP 45640, Jalisco, México

**Keywords:** AFLP, *Fusarium solani*, *in vitro* reproduction, PR proteins, resistance variability

## Abstract

Agave (*Agave tequilana* var. azul) is considered a crop with low genetic diversity because it has been propagated vegetatively for centuries for commercial purposes, and consequently, it could be equally susceptible to pests and diseases. However, the present study employs plant material derived from field-grown plants exhibiting phenotypic variability in susceptibility to agave wilt. The offshoots from rhizomes of these plants were reproduced *in vitro* and classified as potentially resistant or susceptible. Amplified fragment length polymorphism analysis confirmed wide genetic differences among individuals, but these differences were not correlated with the observed phenotypic variability in resistance. Propagated plantlets were inoculated with *Fusarium solani* in two time-lapse confrontations for 72 h and 30 days. The early biochemical response showed statistically superior levels in the accumulation of shikimic acid, phenolic compounds, and chitinase activity in potentially resistant plantlets. There was an inverse correlation of these early biochemical responses and salicylic acid and the incidence of diseased root cells in isogenic plantlets in the 30-day confrontation with *F. solani*, suggesting that these activities and accumulation of molecules were primordial in the defence against this pathogen.

## Teaser

*Agave tequilana*var. azul has been propagated vegetatively for centuries for commercial purposes, and consequently, is believed to be uniformly susceptible to diseases. However, the present study employs plants exhibiting apparent variability in susceptibility to agave wilt as well as genetic diversity. These plants were inoculated with the pathogen *Fusarium solani*and the early biochemical response showed differences in the accumulation of defence-associated molecules and in the level of root damage after 30 days of confrontation, suggesting that in the population there exist individuals with the potential to be selected for their resistance to economically important diseases of this crop.

## Introduction

Agave (*Agave tequilana* var. ‘azul’) is a crop plant specifically required in the tequila industry by the Mexican Official Standard NOM-006-2005 (Diario Oficial de la Federación, Secretaría de Gobernación 2006). In 2020, 374 million litres of tequila were produced using the carbohydrates accumulated in 1.407 million tons of mature agave stems (CRT 2018) cultivated over at least 6 years before harvest on around 19 000 ha (SIAP 2021). *Agave tequilana* var. azul as other in *Agave* genera is a monocarpic crop (that flowers only once and then dies). In the agave production process it is therefore necessary to suppress the sexual reproduction by cutting off any inflorescences that are formed, in order to suspend its growth and prevent that the accumulated carbohydrates in the stem being invested in reproduction. Harvest (called ‘jima’) is the end of production cycle, because the stems are cut at soil level, when have grown enough and increased their fructans stock. For this reason, it has been vegetatively propagated for centuries for commercial purposes, using mainly the rhizome offshoots produced around the agave plants from the second year of this crop cycle. It has therefore been presumed that agave crops had lost genetic diversity with a higher vulnerability to pathogens (Gil-Vega *et al.* 2001; Abraham-Juárez *et al.* 2009; Valenzuela 2011). However, additional reports have shown a significant level of genetic diversity using amplified fragment length polymorphism (AFLP) and inverse sequence-tagged repeat DNA markers (Gil-Vega *et al.* 2006; Torres-Morán *et al.* 2013). This genetic variability may be promising for phenotypic screening in agave populations to identify heritable variants for plant improvement in productivity, fructan content and/or disease resistance (Torres-Morán *et al.* 2010).

Agave wilt is lethal in mature plants, but reports indicate an incidence higher than 50 % in 3-year-old fields (Rubio-Ríos *et al.* 2019) and 82 % in 4-year-old fields (Ramírez-Ramírez *et al.* 2017), with *Fusarium solani* as the main causal agent in the latter. These studies demonstrate the importance of agave wilt; however, in commercial agave fields with a history of high disease incidence, few plants with a healthy appearance stand out among those that are clearly ill, exhibiting promising phenotypic evidence of resistance to this disease. The *F. solani* species complex includes necrotrophic species causing tuber, root and stem rot worldwide (Desjardins 2006). In plant–pathogen interactions, plants protect themselves with induced resistance, increasing the synthesis of compounds associated to defence mechanisms, such as shikimic acid (SHA), *trans*-cinnamic acid (CA), salicylic acid (SA), phenolic compounds from the phenylpropanoid pathway, pathogenesis-related (PR) proteins and phenylalanine ammonia lyase (PAL) activity (Durrant and Dong 2004; Bolton 2009). However, in necrotrophic pathogenesis, the opportunity in the host recognition of the pathogen and the host intensity of the immune responses that control cell death determines host resistance (Mengiste 2012).

In this study, rhizome offshoots from *A. tequilana* mother plants with phenotypic evidence of susceptibility or resistance to agave wilt in commercial fields were compared for genetic diversity with AFLP DNA markers and propagated *in vitro* by meristem culture. This asexual reproduction system has shown low levels of somaclonal variation as meristematic tissue may be preconditioned to maintain low levels of mutation (Díaz-Martínez *et al.* 2012). Plantlets and their descendants were considered individual isogenic lines. The main aim was to correlate the phenotypic variability of these mother plants with the expression in their isogenic lines of compounds associated to defence mechanisms after inoculation with a pathogenic strain of *F. solani* to demonstrate the variability in agave wilt resistance.

The high percentage of variability and the contrasting biochemical responses between potentially resistant and potentially susceptible agave plants to respond to *F. solani* could be used as an additional criterion in future breeding programmes of this crop.

## Materials and Methods

### Sampling and classification of mother plants of *A. tequilana* by phenotypic resistance

**Figure 1.**
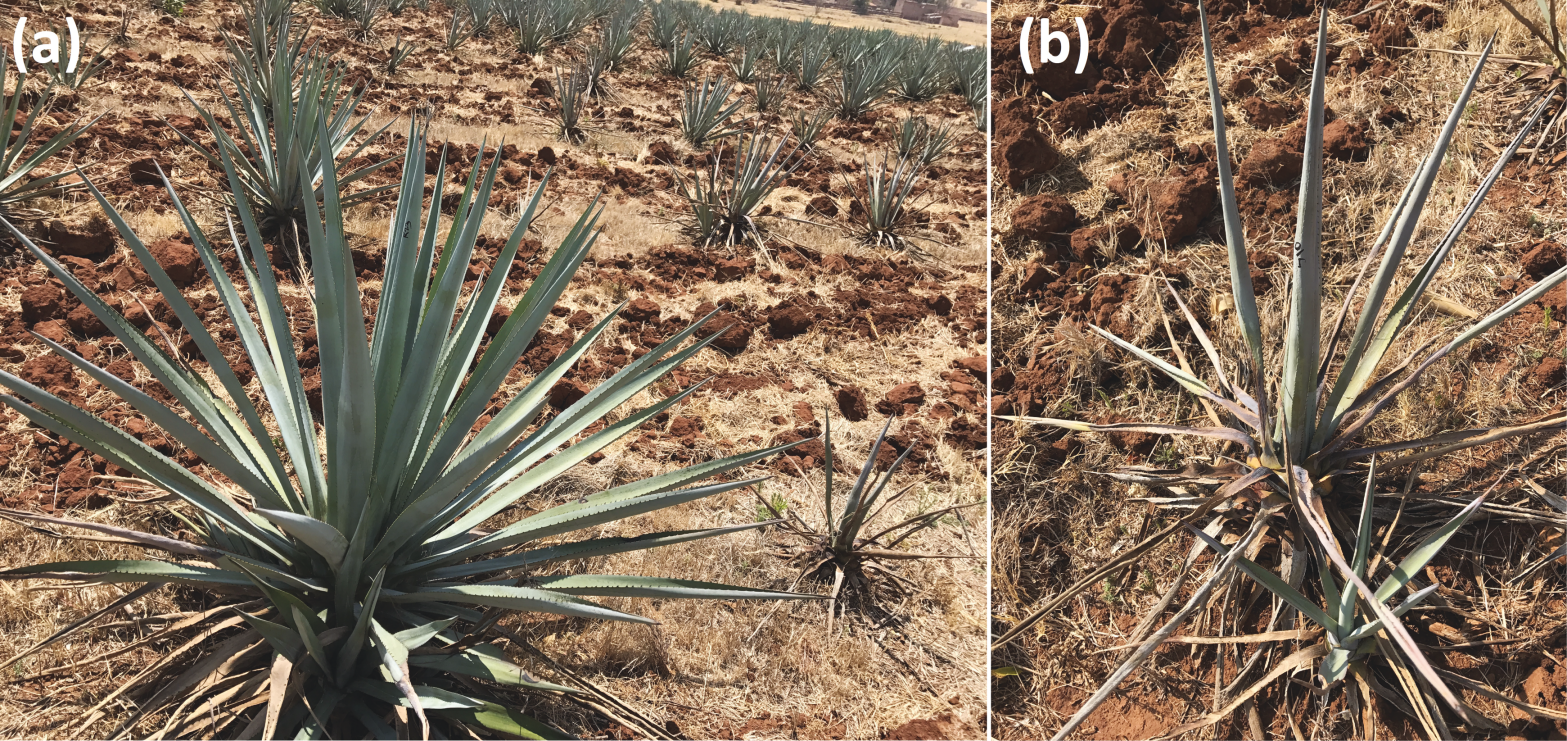
Phenotype of mother plants and their offshoots selected for this work. (A) Mother plant potentially resistant, healthy, with neighbouring mother plants that are clearly diseased. (B) Mother plants wilt with offshoots that are apparently healthy.

In 2011, rhizome offshoots of surviving agave mother plants were collected from different fields with a high incidence of agave wilt disease, to be transplanted in the ‘Genetic Reservoir Area’ of the Tequila Regulatory Council (CRT) in Acatic, Jalisco, Mexico, where previous severe epidemic cycles of this disease were developed. Three years later, rhizome offshoots from these ‘new mother plants’ were donated by the CRT to carry out this work and classified into two phenotypic categories, 33 offshoots were selected as potentially resistant plants (PR plants) based on the healthy appearance of the mother plants in relation to clearly diseased neighbouring plants and 33 offshoots as potentially susceptible plants (PS plants) since their mother showed clear symptoms of agave wilt (Fig. 1). These offshoots were used for *in vitro* multiplication to obtain isogenic lines.

### *In vitro* propagation of offshoots and acquisition of isogenic lines

Offshoots were carefully washed with high-pressure water. The leaf tissue was frozen at −80 °C for DNA extraction. The head containing the meristematic zone was disinfected in a 2 % NaOCl solution and rinsed in sterile distilled water. The edges were removed, and the meristematic zone was cut into 16 equal cubes, soaked in a 0.5 % NaOCl sterile solution for 5 min, rinsed three times with distilled sterile water and plated in Petri dishes with modified MS media (Murashige and Skoog 1962) containing 0.6 mg L^−1^ IAA and 9 mg L^−1^ BAP; Díaz-Martínez *et al.* (2012) reported that this is a reproduction methodology with no variation after four generations. The Petri dishes were incubated in a growth chamber at 25 °C ± 1 and a photoperiod of 16 h. After 4 weeks, the shoots were separated and transplanted to medium with 3 g L^−1^ of activated charcoal in transparent, sterile plastic cups with a lid. The rooted plantlets were transferred to pre-adaptation media, consisting of vermiculite and the non-organic elements used in the previous media. For 4 weeks, the plantlets were gradually conditioned to finally be transferred to a pot with sterile PRO-Mix until inoculation.

### Genetic diversity

To determine the genetic diversity in agave PR and PS plants, AFLP analysis was performed on their offshoots. DNA was extracted according to the Promega (2019) Wizard™ genomic DNA purification kit protocol from disinfected leaf tissue. Aliquots of 2 µL were used for DNA quantification with NanoDrop 8000, and the concentration was standardized to 60 ng μL^−1^ in each sample.

Analysis of AFLP was performed according to Vos *et al.* (1995). Selective amplification of two sets of duplex PCR was run with the combination of three selective bases in the second amplification using *Mse*I + CTT and *Eco*RI primers with three selective bases and ACA, ACT, AGA and AGC at the 3ʹ end and fluorescently labelled with PET, NED, VIC and FAM, respectively, at the 5ʹ end. In a 96-well microplate containing 5.5 µL formamide and 0.5 µL GeneScan 500 LIZ™ dye Size Standard (Applied Biosystems), 2 μL of each selective amplification product of the different combinations of primers were added and sent to LabSerGen in LANGEBIO (Irapuato, Mexico) to be separated in a 96-capillary 3730xl DNA Analyser (Meudt and Clarke 2007). The obtained electropherograms were analysed using Geneious Software v.1.0, and amplified fragments with lengths between 50 and 500 bp were scored to form a presence–absence matrix with a resolution of 1 pb. The AFLP data analysis was carried out in NTSYSpc v.2.21 software using the Dice coefficient to obtain dendrograms.

### Plant–pathogen inoculation

Strain ‘G’ of *F. solani*, pathogenic to *A. tequilana* var. azul (Ramírez-Ramírez *et al.* 2017), with GenBank accession numbers MK027272 for 18S SSU and KU878139 for ITS1-5.8S-ITS2 sequences, was grown on potato dextrose agar in Petri dishes for 7 days to produce inoculum. Suspension of produced conidium was agitated for 12 h in liquid minimum media (Okon *et al.* 1973) to force pre-germination and improve its capacity and speed to infect.

For the 72-h confrontation and the early biochemical response evaluation, plants with similar size and newly developed radicular system were selected. A factorial design was carried out with the combination of two categories (independent variables): phenotype (PR and PS levels), and inoculation (inoculated and non-inoculated levels). Two plants of four isogenic lines were selected for each phenotype (PR7, PR12, PR18, PR36 and PS9, PS11, PS14, PS15), one for each level in the inoculation category. In other words there were four replicate plants in each of the four combinations of phenotype and inoculation treatment. Treatments were compared for their early biochemical responses in the dependent variables PAL, chitinase, peroxidase and β-1,3-glucanase activities, and the SHA, CA, SA and total phenolic compounds concentration.

Sampling was destructive and subsamples were taken according to the tissue availability in each plant.

Individual agave plants were inoculated in the pots with three aliquots of 1 mL 4 × 10^5^ pre-germinated microconidia per mL suspension of *F. solani* ‘G’ strain, added at 3 cm depth to the substrate in three different equidistant points next to the roots. Additionally, according to the intensity in the responses in the previous bioassay (higher in PR plants and lower in PS plants), a second group with less isogenic lines (PR7, PR12, PR36 and PS9, PS11, PS14) were inoculated or non-inoculated and selected for a bioassay with longer confrontation time of 30 days, in order to determinate microscopically, the early signs of root damage.

### PR proteins and PAL activity

To determine the systemic induction of PR proteins and PAL activity, the total proteins were extracted from ground frozen leaf tissues, as described by Zheng and Wozniak (1997), by adding 1 mL cold 50 mM sodium acetate buffer (pH 5.5) to 0.5 g (FW) ground frozen tissue in a 2 mL microcentrifuge tube and incubating them at 4 °C for 5 min at 150 rpm. Samples were centrifuged at 13,500 rpm for 10 min at room temperature. The supernatants were transferred to new microcentrifuge tubes and stored at −70 °C until use. The total protein was quantified according to Bradford (1976) using bovine serum albumin to generate a standard curve. Chitinase, peroxidase, and β-1,3-glucanase activity were assayed according to Reissig *et al.* (1955), Bestwick *et al.* (1998), and Zheng and Wozniak (1997), respectively. PAL enzyme extracts were obtained according to Matros *et al.* (2006), and their activity was measured by CA formation (Koç and Üstün 2012).

### SHA, CA, SA and total phenolic compound concentrations in the roots

Shikimic acid was extracted from powdered dried roots according to Gomes *et al.* (2015). Root extractions of SA and CA were made according to Verbene *et al.* (2002). In all cases, the final recovered product was dissolved in 1 mL absolute methanol and filtered through a 0.45-µm PES filter.

The samples were spiked with a known amount of standard before HPLC analysis. Individual quantification of CA, SA and SHA was performed using the Dionex ICS-5000 HPLC system (Thermo Scientific) equipped with an autosampler (AS-AP) and a photodiode array detector (DAD, Ultimate 3000). Data analysis was carried out using Chromeleon 7.0 software. The optimal wavelength of the molecules was determined by UV spectral scanning, which resulted in 210 nm for SHA, 302 nm for SA and 280 nm for CA. Chromatographic analysis of the CA and SA was performed using an Acclaim 120 C18 column (5 µm, 46 × 150 mm, Thermo Fisher Scientific). The mobile phase was optimized as a mixture of a 3 % aqueous acetic acid solution with methanol (60:40 for SA and 40:60 for CA) at 0.6 mL min^−1^, with an injection volume of 15 µL. For SHA quantification, a C18 Luna column NH_2_ (2.6 µm, 4.6 × 150 mm, Phenomenex) equipped with a security guard column was used. The mobile phase consisted of a mixture of KH_2_PO_4_ 10 mM (pH 4.2) and methanol 50:50 at a flow rate of 0.6 mL min^−1^. The metabolites were quantified by correlating the peak area with the calibration curves of high-purity analytical standards. Calibration curves were made at concentrations from 1 to 20 μg mL^−1^ in each case.

The total phenolic compounds were extracted from frozen samples of ground root mixed with 50 % methanol and sonicated for 30 min. The mixture was centrifuged 10 min at 1400 × *g* and the collected supernatant was used to measure the total phenolic compounds by enzymatic method (Stevanato *et al.* 2004) using a calibration curve with catechol (Sigma, USA). Phenols content was expressed as catechol equivalent (CE), μM CE g^−1^ FW.

### Root necrotic cell incidence

The incidence of root rot was evaluated in plantlets after 30 days of confrontation with *F. solani*. In a destructive sampling, five root fragments (2 cm long) from different levels of the root system in each plant were randomly taken. The sections were examined with a compound light microscope (Carl Zeiss®, AxioLab) at 40×. Random digital photographs of at least five microscope fields per root section were obtained using the imaging software ZEN® Blue edition coupled to the microscope. The percentage of incidence of visibly damaged cells in a known number of total cells in each microscope field was recorded for plants inoculated and non-inoculated with *F. solani* in PR and PS plants. The obtained percentage data were modified by angular transformation for statistical analysis.

### Correlation of host defence mechanisms with the incidence of diseased root cells

Pearson correlation analysis was performed with the average of both, incidence percentage of diseased root cells after 30 days of confrontation and the early biochemical defence responses (SHA, SA, CA, PAL, PRs activities and phenolic concentration) registered at 72 h in plantlets of some isogenic lines (PR7, PR12, PR36 and PS9, PS11), to identify the main defence mechanism explaining the incidence of diseased root cells caused by pathogenic *F. solani*.

### Statistical analysis

The content of compounds associated to defence mechanisms presented is the average of at least four replicates, processed independently. Data were analysed by two factors in a general linear model and a Duncan test, using SAS® ver. 8.0 statistical software.

## Results

### *In vitro* reproduction of agave plants

**Figure 2.**
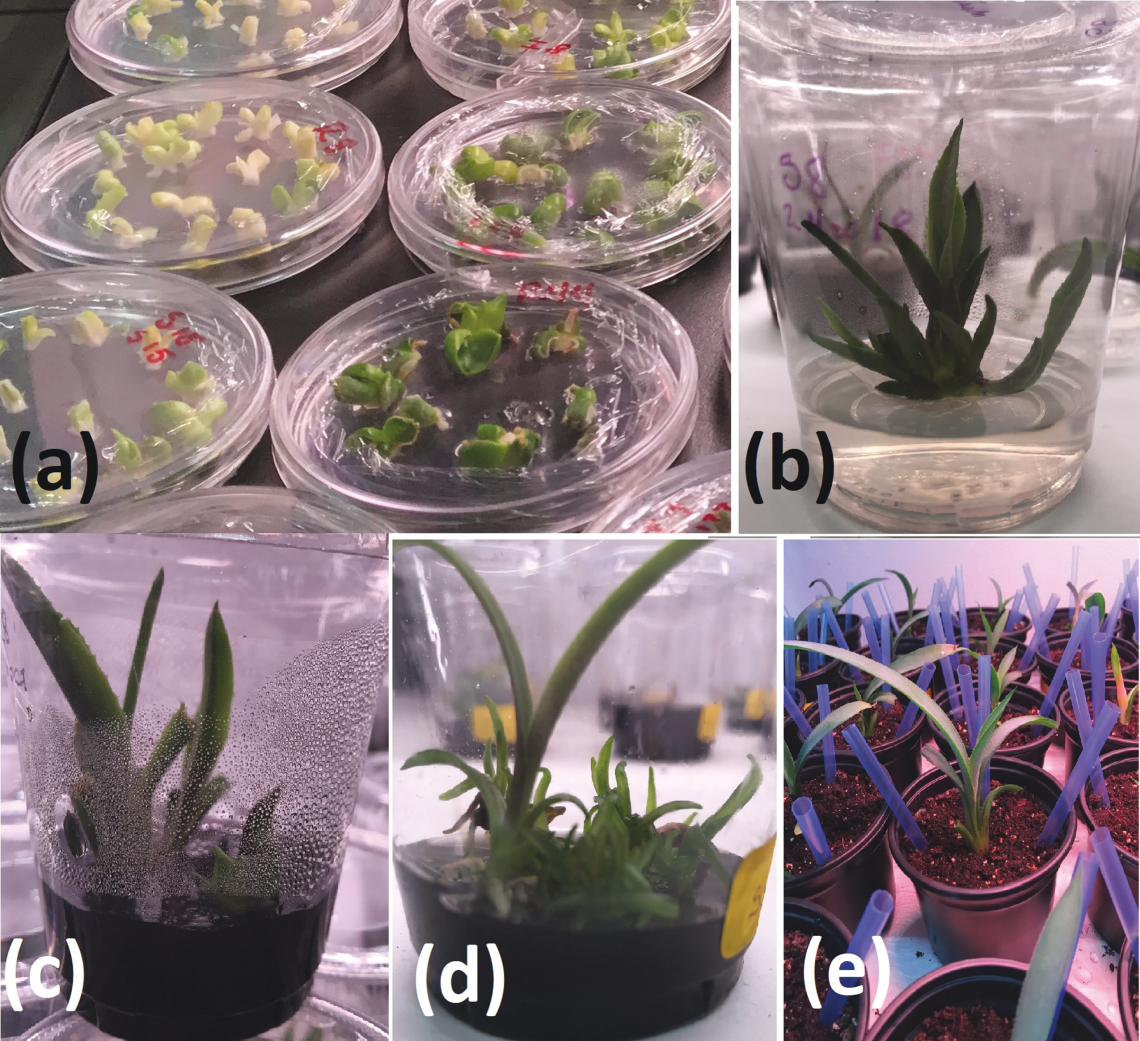
*In vitro* reproduction of *Agave tequilana* var. azul plantlets. (A) Excision of the 16 fragments from the meristematic tissue. (B) Multiplication stage. (C and D) Differences in the capability of shoot formation by an individual plantlet. (E) *Ex vitro* adaptation stage before inoculation.

Shoot production in *in vitro* culture was highly variable for each agave offshoot, since some offshoots generated zero shoots and other generated up to 24 with a mean of 8. However, in the latter case, when shoots were generated, they also yielded new ones during the propagation stage, and some new offshoots were visible during the *ex vitro* adaptation stage (Fig. 2). Díaz-Martínez *et al.* (2012) reported that this is a reproduction methodology with no variation after four generations. The exact same reproduction process was applied in all the individuals to ensure that all the statistical differences in quantified defence mechanisms were related with genotype in spite of the reproduction method.

### AFLP analysis

**Figure 3.**
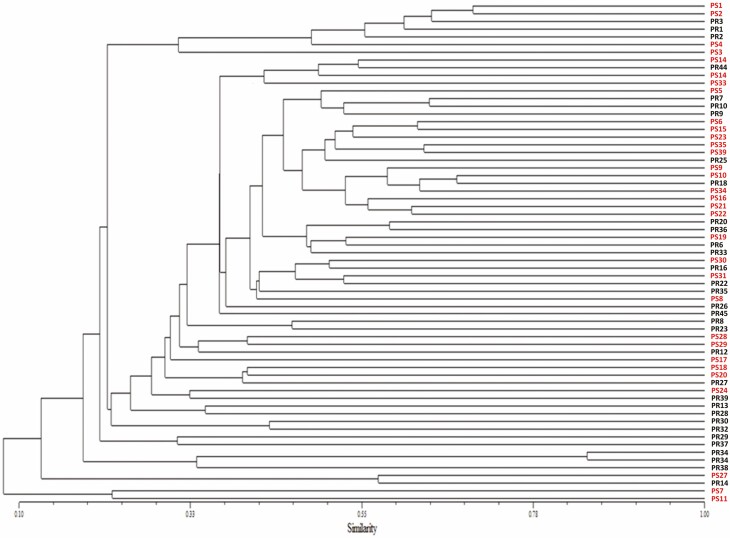
Amplified fragment length polymorphism (AFLP) similarity dendrogram of 86 agave (*Agave tequilana* var. azul) offshoots from mother plants with presumably resistant (R) or susceptible (S) phenotypes. Data obtained after amplification with EcoRI + AGC and MseI + CTT primers. Dendrograms were made using NTSys pc2.2 software, the Dice index and the UPGMA clustering method.

Electropherograms were obtained from each AFLP primer combination with amplicons ranging from 60 to 400 bp. The number of amplified fragments resolved as a definition of 1 pb was highly variable in the four primer combinations between offshoots, with an average of 43 % variability, 176 polymorphic markers and 35 unique markers. From each primer combination, four dendrograms were constructed, and overall, genetic variability among specimens was evident without showing phenotype-related clusters, as shown in Fig. 3, with EcoRI + AGC and MseI + CTT primers **[see****Supporting Information 1****]**.

### Systemic PR proteins and PAL activity

**Figure 4.**
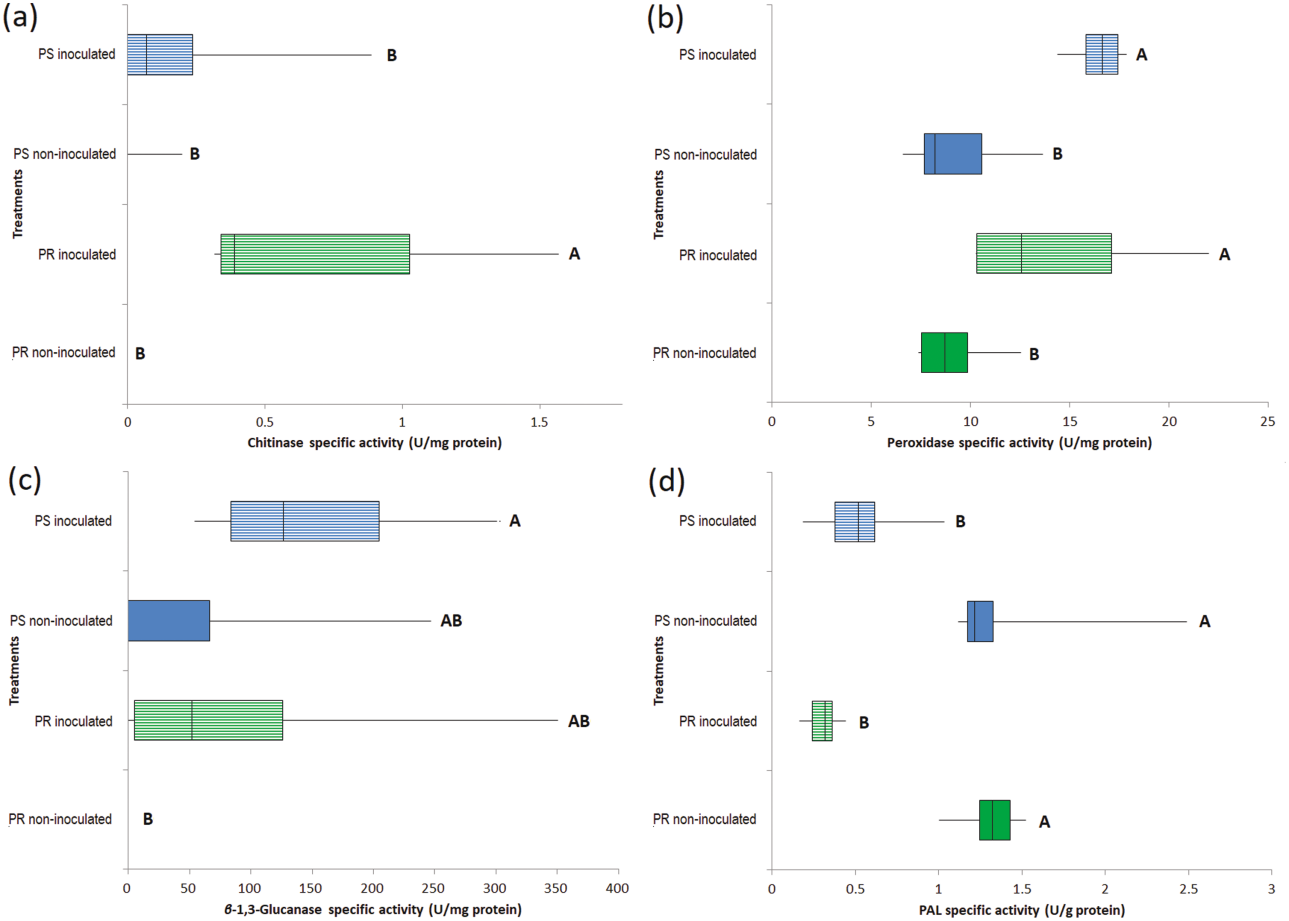
Pathogenic-related proteins and PAL activity in leaf extracts of *Agave tequilana* var. azul plantlets that were potentially resistant (PR) or susceptible (PS), 72 h after inoculation or not with pre-germinated conidia of pathogenic *Fusarium solani* ‘G’ strain in their root system. (A) Chitinase activity; (B) peroxidase activity; (C) β-1,3-glucanase activity; and (D) PAL activity. One unit (U) of chitinase activity equals 1 mg chitin from crab shell hydrolysed per minute per mg of protein. One unit (U) of peroxidase activity was expressed as the difference in absorbance at 490 nm per min after 3 min per mg of protein. One unit (U) of β-1,3-glucanase activity equals 1 mM of D-glucose hydrolysed from laminarin per mg of protein per min. One unit of PAL activity corresponds to 1 µM of *trans-*cinnamic acid produced in 1 min per mg of protein. Treatments sharing the same letter are not significantly different (Duncan *P* < 0.05).

Potentially resistant non-inoculated plantlets did not have chitinase activity; however, in the presence of the pathogen, this activity was induced at a mean level of 0.73 U. Data from PS plantlets showed a smaller difference among the non-inoculated plantlets and those that were inoculated, with an average value less than 0.2 U (Fig. 4A).

In non-inoculated plantlets, the basal peroxidase activity in PR and PS was about 9 U without a significant difference. However, after 72 h, the presence of the pathogen induced a significant increment to 14.4 and 16.4 peroxidase U in both the PR- and PS-inoculated agave plantlets, respectively, but there was no significant difference between them (Fig. 4B).

The activity of β-1,3-glucanase in the PR and PS plantlets at 72 h reached 84.4 and 145.43 U, respectively, however were not significantly superior to those levels observed in the non-inoculated (Fig. 4C). In contrast, the basal 1.32 and 1.21 U of PAL activity were significantly reduced to 0.31 and 0.27 U in leaves, respectively, 72 h after PR and PS plantlets were inoculated with *F. solani* (Fig. 4D) **[see****Supporting Information 2****]**.

### SHA, CA, SA and total phenolic compound concentrations in roots

**Figure 5.**
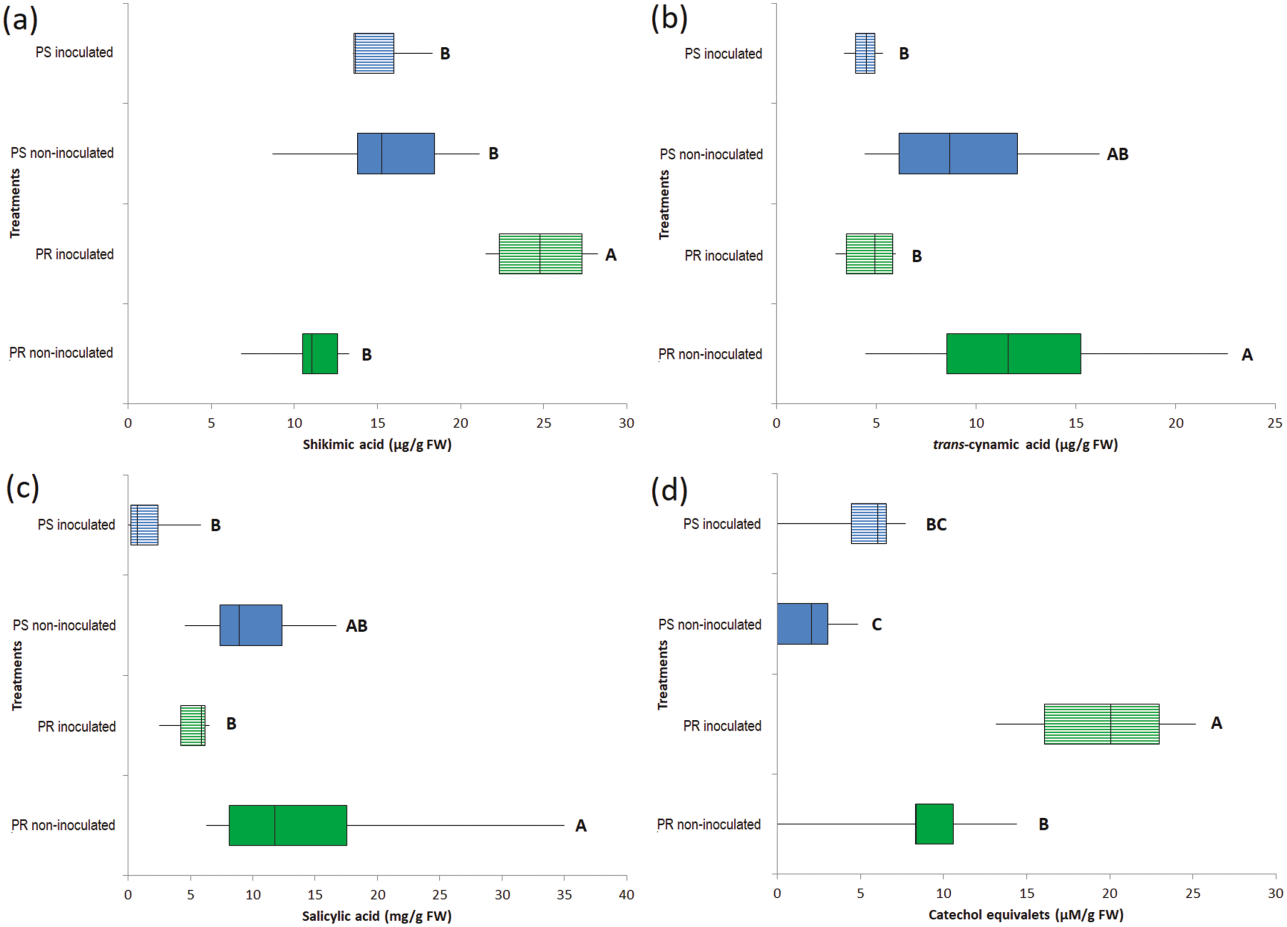
Concentrations of shikimic acid (A), *trans-*cinnamic acid (B), salicylic acid (C) and total phenolic compounds (D) in root extracts of *Agave tequilana* var. azul plantlets that were potentially resistant (PR) and susceptible (PS), 72 h after inoculation or not with pre-germinated conidia of *Fusarium solani* ‘G’ strain. Treatments sharing the same letter are not significantly different (Duncan *P* < 0.05).

When the content of SHA was quantified, the basal concentration of this metabolite was 10.85 µg g^−1^ DW in extracts of PR plantlet roots. This concentration significantly increased to 24.84 µg g^−1^ DW 72 h after inoculation with *F. solani*, as an early response to its pathogenic process. Meanwhile, the baseline SHA level (15.52 µg g^−1^ FW) of PS plantlets remained statistically similar after 72 h of inoculation with pre-germinated conidia of *F. solani* (Fig. 5A).

After 72 h of inoculation, PR plantlets showed a statistically significant reduction in CA to 4.64 µg g^−1^ FW compared to the baseline in non-inoculated (12.5 µg g^−1^ FW); inoculated PS plantlets were statistically similar to non-inoculated ones (Fig. 5B). In contrast, the inoculation of agave plantlets of different isogenic lines with *F. solani* resulted in a reduction of the SA content in both PR and PS plantlets, from their basal level (15.72 and 9.68 µg g^−1^ FW, respectively) to that registered 72 h after inoculation (4.96 and 1.83 µg g^−1^ FW, respectively), as an early response (Fig. 5C).

The basal level of total phenolic compounds in non-inoculated plantlets was significantly different between PR and PS plantlets, with a mean of 8.33 and 1.94 µM CE g^−1^ FW, respectively. After 72 h of inoculation with *F. solani*, PR plantlets had a statistically higher content of phenolic compounds (19.52 µM CE g^−1^ FW) compared to PS plantlets and both non-inoculated control plants (Fig. 5D) **[see****Supporting Information 3****]**.

### Root necrotic cell incidence in agave

**Figure 6.**
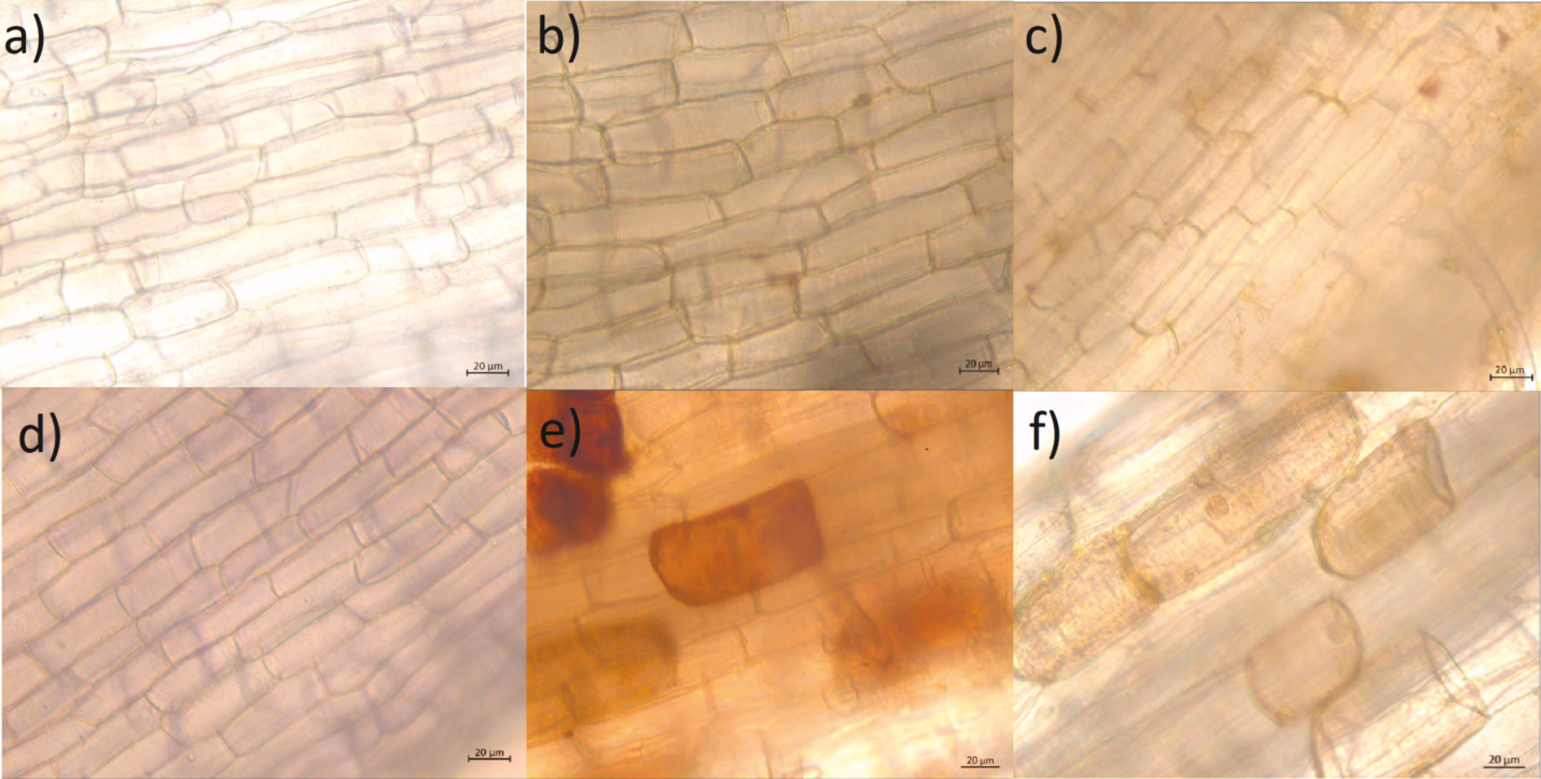
Microscope optic fields with *Agave tequilana* var. azul root cells, 30 days after inoculation with pre-germinated conidia of *Fusarium solani* ‘G’ strain. (A and B) Non-inoculated controls; (C and D) roots of inoculated R plantlets; and (E and F) symptoms in root cells mostly observed in S plantlets.

**Figure 7.**
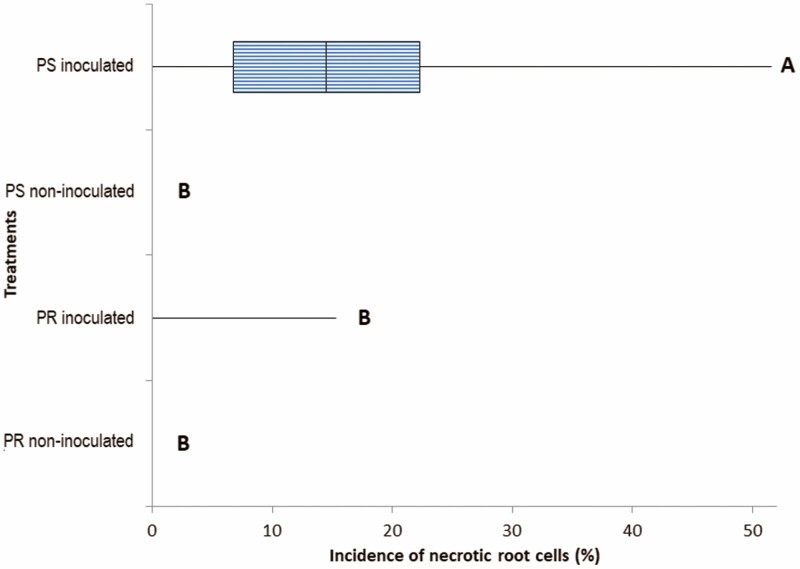
Incidence of root rot cells in *Agave tequilana* var. azul, 30 days after inoculation with the *Fusarium solani* ‘G’ strain in plantlets potentially resistant (PR) and susceptible (PS) and their non-inoculated controls. Plots sharing the same letter are not significantly different (*P* < 0.05).

The interaction between agave plantlets and *F. solani* over 30 days provoked the presence of root rot cells, especially in PS plantlets, which showed the successful necrotrophic parasitism of the strain, as shown in Fig. 6**[see****Supporting Information****]**. Necrotic root cells quantified in the optical fields of the microscope showed a significant difference between treatments. Of the necrotic cells, 20 % were observed in PS plantlets, and there were significantly more compared to the average of 3.2 % intolerant phenotype PR plantlets, which were statistically slightly superior to non-inoculated controls (Fig. 7) **[see****Supporting Information 4****]**.

### Correlation between incidence and early response of defence mechanisms

When the incidence of necrotic root cells of PR and PS plantlets 30 days after inoculation with *F. solani* was correlated with the early biochemical responses after 72 h of inoculation with the same pathogen in plantlets of the same isogenic lines, correlation coefficients of −0.86, −0.68, −0.86 and −0.74 were determined for the content of SHA, SA, chitinase activity and phenolic compounds content, respectively. The activities of β-1,3-glucanase and peroxidase had a correlation of 0.85 and 0.79, respectively, with incidence recorded 30 days after inoculation **[see****Supporting Information—Table S1****]**.

## Discussion

There is a negative impact on the yield of agave crops caused by *F. solani*, the causal agent of agave wilt (Ramírez-Ramírez *et al.* 2017; Rubio-Rios *et al.* 2019), suggesting that it is accentuated because of the low genetic diversity in *A. tequilana* var. azul. However, evidence of genetic variability in this vegetatively propagated crop (Gil-Vega *et al.* 2006; Torres-Morán *et al.* 2013) and the presence of healthy agave plants among other wilted ones in commercial fields support the existence of resistance variability. This study demonstrated, for the first time, differences in the early biochemical responses 72 h after inoculation with the pathogenic *F. solani* strain ‘G’ in those plantlets propagated *in vitro* from mother plants with phenotypic resistance (PR plants) from commercial agave fields. For the success of the plant immune system, early recognition of the pathogen and a timely trigger of defence responses are critical (Vallad and Goodman 2004).

The increase in SHA concentration in PR plants inoculated with *F. solani* is evidence of modifications in their primary metabolism to provide chorismate, a precursor of the aromatic amino acids for an increment in protein synthesis, or to support the increased flux of carbon to the secondary metabolism necessary for a faster defence response (Bolton 2009). The level of PAL and CA, initial products of the phenylpropanoid pathway, was significantly reduced at the 72-h evaluation. For PAL, this likely occurred because increased activity is registered at a maximum level a few hours after inoculation due to *de novo* induction and the subsequent decrease in activity; this time frame might be variable according to the host–pathogen system (Zhang and Liu 2015). The reduction of CA in both PR and PS plantlets could be explained due to its nature as a transient molecule; it is rapidly channelled into optional branched routes of the phenylpropanoid pathway and induced as a defence against the pathogen (Shuab *et al.* 2016).

The decrease in SA observed in both phenotypes as an early response in infected roots could be related to the systemic translocation to the non-infected distal parts of the plant (Maruri-López *et al.* 2019) to promote the defence-related transcription (Lim *et al.* 2020), including the expression of certain PR proteins (Glazebrook 2005). In this study, chitinase activity was differentially induced in PR genotypes, suggesting that this activity is primordial in the defence against *F. solani*. The induction of chitinase isoforms by the presence of *F. oxysporum*, as a biotic agent, has also been demonstrated in *A. tequilana* (Sierra-Gómez *et al.* 2019).

The content of SHA, SA, phenolic compounds and chitinase activity found in agave plantlets 72 h post-inoculation with *F. solani* were negatively correlated with the incidence of diseased root cells of plantlets of the same isogenic lines confronted by 30 days (interpretation of this correlation should consider that only five data points were used). Despite of this, results are in agreement with Santos-Sánchez *et al.* (2019), who reported that a faster and higher production of SHA is key for the subsequent biosynthesis of aromatic amino acids and phenolic compounds necessary for an efficient defence response. However, in the case of PS plants, the evidence of cell death 30 days after inoculation with *F. solani* is an early indicator of successful infection (van Kan 2006), considering that this pathogen takes 6 months to necrotize the majority of the root of *A. tequilana*, using a similar inoculation (Ramírez-Ramírez *et al.* 2017). Mengiste (2012) reported that when a host fails to constrain cell death in an interaction with a necrotrophic pathogen, the plant dies.

Although this study showed an increase in the levels of peroxidase activity in both phenotypes as an early response to *F. solani* inoculation, the high phenolic content found only in PR plantlets with less root cell damage marks the difference. An increase in peroxidase activity during incompatible plant–pathogen interactions is associated with a reduced susceptibility to *F. solani*, for example, because lignification of upper roots reported in soy (Lozovaya *et al.* 2004) and suberization in potato tubers (Lulai and Corsini 1998) act as mechanical barriers. Additionally, some hosts of *F. solani*, such as potato and soy, produce the phytoalexins rhisitin, glyceollin, respectively, as resistance phenolic molecules, which may reduce their susceptibility to this common pathogen of plants (Coleman 2016). The combination of an increment in phenolic compounds and peroxidase activity in agave plants, without evidence of damage to their root cells, suggests that one of the routes taken by the potentially resistant agave plant to defend itself against *F. solani* was mediated by SA signalling (Bawa *et al.* 2019), but further studies should be conducted to confirm this hypothesis.

The ability of AFLP markers to detect wide genetic differences among individuals was demonstrated here for *A. tequilana* var. azul in 66 specimens. This high percentage of natural variability in number of polymorphic and unique markers found by AFLP analysis in this work was superior to the variability induced by ionizing radiation with gamma Co^60^ in this crop (Hernández-Hernández 2004; Ángeles-Espino *et al.* 2020). However, “molecular unique markers generated by AFLP associated to the more resistant and the more susceptible plants found in this work still need to be identified”, as a simpler and faster strategy to detect elite isogenic lines as other works have demonstrated (Dwivedi *et al.* 2002; Jiang *et al.* 2007). Additionally, the contrasting biochemical responses presented here are evidence of the natural functional variability in *A. tequilana* var. azul. To counteract to pathogens, resistant plants should modify several metabolic pathways for the synthesis of different antimicrobial metabolites. This work demonstrates changes in some of them; however, this must be complemented with analysis such as metabolomics studies based on gas or liquid chromatography coupled to mass spectrometry and/or Nuclear Magnetic Resonance (NMR) in addition to quantitative measurements and the time dynamics of metabolic responses to the challenge with isogenic lines of PR elite and PS agave plants with the pathogenic fungi *F. solani* (Chen *et al.* 2019). Additionally, microarray transcriptomic analysis could be used to determinate the gene expression profile of PR and PS plants (Chen *et al.* 2020) and genotyping platforms could be used to identify single-nucleotide polymorphism that could be used as genetic markers useful in breeding programmes (Wen *et al.* 2014; Rasheed *et al.* 2017).

Selection of resistant varieties is considered to be the best way of solving the wilt disease problem in other crops. At the moment, there is no availability of *A. tequilana* germoplasm with proved resistant to *F. solani.* Conventional breeding programmes are a very slow strategy, and *A. tequilana* takes from 6 to 8 years to reach sexual reproduction; therefore, it is more convenient initially the use of *in vitro* culture tissue to reproduce those plants selected with genetic resistance to agave wilt and, according to the report of Díaz-Martínez *et al.* (2012), the reproduction method employed in this work avoids high level of somaclonal variation unlike the found in other crops after vegetative reproduction (Hwang and Ko 2004). Further works with longer confrontation times should be focused on the selection of the elite isogenic lines, considering their resistance to *F. solani*.

## Supporting Information

The following additional information is available in the online version of this article—

Supporting Information 1. Amplified fragment length polymorphism (AFLP) matrix.

Supporting Information 2. Enzymatic activities.

Supporting Information 3. Defence compounds.

Supporting Information 4. Root rot cells incidence.

Table S1. Pearson correlations.

plac027_suppl_Supplementary_Materials

## Data Availability

The data are provided as **Supporting Information**.

## Notes

**Natural History and Conservation. Chief Editor**: F. Xavier Picó
