# Supplementary material for: Asexually propagated Agave tequilana var. azul exhibits variation in genetic markers and defence responses to Fusarium solani
Source: AoB Plants. 2022 Jun 8;14(3):plac027. doi: 10.1093/aobpla/plac027 (PMC9246091; doi:10.1093/aobpla/plac027)
Supplement: plac027_suppl_Supplementary_Materials [file plac027_suppl_supplementary_materials.zip › plac027_suppl_Supplementary_Table_S1.pdf]

Table 1S. Pearson correlations between early biochemical responses and the incidence of diseased root cells in iseogenic plantlets of the 30-days confrontation with *F. solani.* Data are the average of several determinations in each of the five isogenic plantlets presented.

Sample name	Incidence (%)	PAL activity (U)	Shikimic acid (µg /g DW)	*t* -cinnamic acid (µg /g FW)	Cathecol eq. (µM / g FW)	Peroxidase activity (U)	Chitinase activity (U)	β-1,3-glucanase activity (U)	Salicylic acid (mg/g FW)
PR inoculated	2.89	0.25	27.62	5.64	17.29	15.03	0.67	81.18	5.87
PR inoculated	0.00	0.41	22.61	2.96	13.15	10.32	0.95	65.02	2.53
PR inoculated	2.00	0.25	21.54	5.34	23.85	10.28	0.39	0.00	6.50
PS inoculated	12.24	0.38	15.90	4.92	4.03	16.68	0.25	134.27	2.03
PS inoculated	17.79	0.70	13.67	3.38	7.70	16.04	0.08	152.87	1.24
Pearson correlation		0.78	**-0.86**	-0.22	**-0.74**	0.79	**-0.86**	0.85	**-0.68**
